# Early Outcomes of Real‐World Aortic Valve Replacement With RESILIA Tissue in the Chinese Population

**DOI:** 10.1002/clc.70347

**Published:** 2026-06-15

**Authors:** Haitao Xu, Yang Yan, Xin Chen, Jiangang Wang, Hansong Sun

**Affiliations:** ^1^ Department of Cardiovascular Surgery Fuwai Hospital, Chinese Academy of Medical Sciences and Peking Union Medical College Beijing China; ^2^ Department of Cardiovascular Surgery The First Affiliated Hospital of Xi'an Jiaotong University Xi'an China; ^3^ Department of Cardiovascular Surgery Nanjing First Hospital Nanjing China; ^4^ Valve and Atrial Fibrillation Surgery Center, Beijing Anzhen Hospital, Capital Medical University Beijing China

**Keywords:** aortic valve replacement, bioprosthetic valve, real‐world data, safety, valve function

## Abstract

**Background:**

Aortic valve replacement (AVR) with the INSPIRIS RESILIA aortic valve (Edwards Lifesciences, Irvine, CA) has established durability and promising outcomes. However, real‐world safety and functional performance data remain limited. This study reported early outcomes in the Chinese population.

**Methods:**

This prospective, multicenter, single‐arm, post‐market, real‐world study enrolled patients scheduled for elective AVR using the study valve. Safety outcomes included all‐cause death, study valve‐related death, structural valve deterioration (SVD), non SVD, reoperation on the study valve, major bleeding, and thromboembolic events. Effectiveness outcomes included valve hemodynamic performance and New York Heart Association (NYHA) class.

**Results:**

A total of 250 patients underwent study valve implantation, with 238 completing 1‐year follow‐up (mean follow‐up duration: 448.2 ± 162.50 days). The mean age was 58.5 ± 9.17 years, with 11.4% undergoing concomitant aortic root/annular enlargement. The 1‐year freedom from all‐cause death and freedom from reoperation on the study valve were 98.0% and 99.2%, respectively. All deaths were assessed as unrelated to the study device. Incidence of complications included thromboembolic events (2.4%), major bleeding (4.4%), endocarditis (0.4%), and new permanent pacemaker implantation (0.4%) at 1 year. Two patients (0.8%) required reoperation on the study valve. No SVD was observed. At 1‐year post‐operation, no severe aortic regurgitation (AR) occurred, while 1 patient (0.4%) developed moderate AR. Three patients (1.3%) had mild paravalvular leakage. Functional status improved with 100% of patients having a NYHA Class I/II at 1 year.

**Conclusion:**

INSPIRIS RESILIA aortic valve shows favorable 1‐year safety profiles and stable hemodynamics in Chinese patients.

## Summary

- In this real‐world study of 250 Chinese patients, the INSPIRIS RESILIA aortic valve demonstrated excellent 1‐year safety outcomes, with a 98.0% freedom from all‐cause mortality and no occurrences of structural valve deterioration. The incidences of major bleeding (4.4%) and thromboembolic events (2.4%) were low.
- The valve demonstrated stable hemodynamics at 1‐year follow‐up. A significant improvement in functional status was observed, with 100% of patients achieving New York Heart Association Class I or II.
- The study population had a higher proportion of concomitant root enlargement procedures compared to prior Western trials, suggesting a trend toward more root enlargement among Chinese surgeons, which may be beneficial for long‐term outcomes and comprehensive lifelong management.

## Graphical abstract

In this real‐world study of 250 Chinese patients scheduled for elective aortic valve replacement, the INSPIRIS RESILIA aortic valve demonstrated favorable 1‐year safety, with a 98.0% rate of freedom from all‐cause mortality and no observed structural valve deterioration, alongside stable hemodynamic performance.

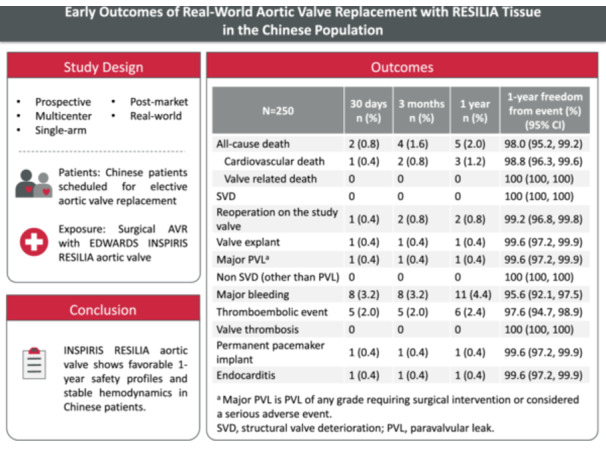

## 1 Introduction

The global burden of valvular heart disease (VHD) is rising markedly, attributed to enhanced survival rates and population aging [1, 2]. VHDs contribute substantially to mortality and morbidity and reduced quality of life [3]. In China, VHDs affect approximately 25 million individuals, with aortic valve diseases (AVDs) constituting a substantial subset [4]. Concurrently, greater awareness and the expanding availability of echocardiography facilitate earlier diagnosis [2]. This evolving epidemiological landscape demands the implementation of comprehensive life‐cycle management strategies, including enhanced valve durability.

Conventional bioprosthetic valves commonly require glutaraldehyde processing to increase stability [5, 6]. However, residual aldehyde groups can trigger calcification, compromising long‐term outcomes due to structural valve deterioration (SVD) [6]. A meta‐analysis of bioprosthetic valve replacement revealed 15‐year SVD‐free survival rates of 61.6%−88.9%, which declined to 25.2%−56.0% at 20 years [7]. Conversely, mechanical valves provide superior long‐term durability but require lifelong anticoagulation treatment and carry reoperation risks [8]. Increasing life expectancy and escalating perioperative risks in aging populations demand next‐generation innovations.

To address this challenge, the RESILIA technology was developed to mitigate calcification and improve durability by targeting three key pathogenic mechanisms in conventional bioprosthetic valves: phospholipid content, residual free aldehyde groups, and secondary aldehyde exposure [9]. The tissue treatment also allows dry storage of the valve. The INSPIRIS RESILIA aortic valve (Edwards Lifesciences, Irvine, CA) features an expandable stent frame to optimize potential future valve‐in‐valve replacement [10]. Accelerated wear testing simulating 50 years of function (2 billion cycles) demonstrated preserved hemodynamic performance, leaflet kinematics, and flow characteristics [11]. Clinically, the multinational COMMENCE trial reported 100% freedom from SVD at 5 years [12] and 99.3% at 7 and 8 years [13, 14], suggesting excellent durability and hemodynamic performance.

While the COMMENCE trial established the valve's long‐term safety, its generalizability to real‐world populations with greater disease severity and more complex concomitant interventions requires investigation. Additionally, Chinese patients with AVD exhibit distinct anatomical characteristics [15, 16]. This post‐market, real‐world study aimed to evaluate the safety and functional outcomes of the INSPIRIS RESILIA aortic valve in the Chinese population.

## 2 Methods

### 2.1 Study Design and Patients

This prospective, multicenter, single‐arm, post‐marketing, real‐world study included a 5‐year follow‐up. Patients were assessed at baseline, discharge, 30 days (via telephone follow‐up), 3 months, and annually thereafter. The present analysis focuses on effectiveness and safety outcomes at the 1‐year follow‐up.

Patients were enrolled across 12 centers in China. Eligible patients were adults scheduled for elective aortic valve replacement (AVR) (native or prosthetic). Concomitant procedures, including coronary artery bypass grafting (CABG) or ascending aortic replacement, were permitted. Exclusion criteria included: (1) contraindications to valve implantation; (2) planned multiple valve replacements or repairs; (3) active endocarditis or myocarditis before the scheduled AVR; (4) life expectancy < 12 months; or (5) current or recent (within 6 weeks prior to the procedure) participation in another clinical trial.

The study adhered to the Declaration of Helsinki and local regulations. Ethical approval was obtained from the institutional review board at each participating center (Supporting Information Materials). Written informed consent was obtained from all patients. The trial was registered at ClinicalTrials.gov (NCT05404880).

### 2.2 Study Device and Surgical Procedures

The INSPIRIS RESILIA aortic valve (model 11500 A, Edwards Lifesciences, Irvine, CA, USA) incorporates the RESILIA technology. Available sizes included: 19, 21, 23, 25, 27, and 29 mm.

Implantation was restricted to surgeons with appropriate training in AVR. The determination of surgical approach and implantation technique, including full sternotomy, mini upper sternotomy, and right thoracotomy, was at the surgeon's discretion. All procedures adhered to standardized surgical protocols [17]. Patients were maintained on antiplatelet therapy following clinical guidelines unless contraindicated [18]. However, the final decision to start and stop the antiplatelet and/or anticoagulation regimens was at the surgeon's discretion.

### 2.3 Outcomes

The safety outcomes comprised all‐cause death, study valve‐related death, SVD, non SVD, reoperation on the study valve, major bleeding, and thromboembolic events. These adverse events were defined in accordance with standard criteria [18, 19].

The effectiveness outcomes were valve hemodynamic performance and New York Heart Association (NYHA) functional class. Valve hemodynamics were evaluated using transthoracic echocardiography, assessing parameters including mean and peak transvalvular pressure gradients, effective orifice area (EOA), EOA index, and valve regurgitation (including paravalvular leakage [PVL]).

### 2.4 Statistical Analysis

Accounting for potential attrition from loss to follow‐up, withdrawal, and other unanticipated dropouts, a maximum of 250 patients requiring AVR were estimated for implantation of the study valve. Three analysis sets were defined: enrolled, valve‐implanted, and 1‐year study completion populations.

The enrolled population comprises patients who voluntarily participated in the study, signed the informed consent form, and initiated the AVR procedure (defined as entry into the operating room). The implanted population comprised the patients who had the study valve successfully implanted and retained upon leaving the operating room. The study completion population encompasses patients who completed the 1‐year postoperative study follow‐up.

Categorical variables are presented as *n* (%). Continuous variables are presented as means ± standard deviations (SD). The Kaplan−Meier method was used to estimate all safety outcomes with 95% confidence intervals (CIs), and the corresponding curves were plotted. All statistical analyses were performed using SAS 8.3.

## 3 Results

### 3.1 Baseline Characteristics

**Figure 1:**
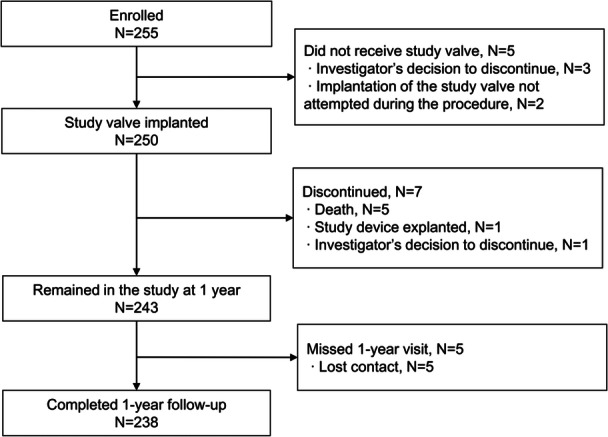
Flowchart.

Between July 2022 and March 2025, 255 patients were enrolled (i.e., the enrolled population), of whom 250 received the valve implant (i.e., the valve‐implanted population), and 238 completed the 1‐year follow‐up (i.e., study completion population) (Figure 1 and Supporting Information S1: Figure 1). The mean follow‐up was 448.2 ± 162.50 days. In the enrolled population, the mean age was 58.5 ± 9.17 years. Most patients were in NYHA Class II (39.6%) or III (49.8%). Degenerative (60.0%), congenital (28.2%), and rheumatic (6.7%) were the most common etiologies. The types of valvular dysfunction requiring AVR included pure insufficiency (54.9%), stenosis with insufficiency (34.1%), stenosis alone (9.4%), and other causes (1.6%) (Table 1).

**Table 1:** Baseline characteristics of the patients (enrolled population).

Characteristics	Patients (*n* = 255)
Age (years), mean ± SD (min, max)	58.5 ± 9.17 (18, 78)
Sex, *n* (%)	
Male	208 (81.6)
Female	47 (18.4)
New York Heart Association classification, *n* (%)	
I	6 (2.4)
II	101 (39.6)
III	127 (49.8)
IV	6 (2.4)
Body mass index (kg/m²), mean ± SD	25.02 ± 3.36
>30	14 (5.5)
Etiology, *n* (%)	
Degenerative	153 (60.0)
Congenital	72 (28.2)
Rheumatic	17 (6.7)
Dystrophic calcification	6 (2.4)
Remote endocarditis	1 (0.4)
Other etiology	16 (6.3)
Diagnosis for replacement, *n* (%)	
Pure insufficiency	140 (54.9)
Stenosis with insufficiency	87 (34.1)
Stenosis	24 (9.4)
Other diagnoses^a^	4 (1.6)
Cardiovascular comorbidities, *n* (%)	
Aortic insufficiency	234 (92.5)
Mitral insufficiency	177 (69.4)
Tricuspid insufficiency	153 (60.0)
Systemic hypertension	137 (53.7)
Cardiac rhythm abnormalities/conduction disturbances	114 (44.7)
Aortic stenosis	112 (43.9)
Mild	20 (17.9)
Moderate	24 (21.4)
Severe	66 (58.9)
Unknown	2 (1.8)
Peripheral artery/vascular disease	82 (32.3)
Coronary artery disease	60 (23.7)
Hyperlipidemia or hypercholesterolemia	47 (18.5)
Carotid artery disease	44 (17.5)
Pulmonary hypertension	31 (12.3)
Cerebrovascular accident or stroke	16 (6.3)
Congestive heart failure	13 (5.2)
Transient ischemic attack	7 (2.7)
Pulmonary insufficiency	6 (2.4)
Myocardial infarction	4 (1.6)
Endocarditis	4 (1.6)
Cardiomyopathy	3 (1.2)
Mitral stenosis	1 (0.4)

^a^ Other diagnoses include congenital bilobular aorta malformation, aneurysm of the ascending aorta, and aortic valve prolapse and insufficiency.

### 3.2 Procedural Outcomes

In the valve‐implanted population, 46.4% of patients underwent isolated AVR. Concomitant procedures included ascending aortic replacement (28.2%), CABG (14.1%), aortic aneurysm/dissection repair (13.7%), and root sinus enlargement aortic/annular enlargement (11.4%). The mean aortic cross‐clamp time and cardiopulmonary bypass time were 84.8 ± 30.40 min and 118.0 ± 42.14 min, respectively (Table 2).

**Table 2:** Intraoperative and postoperative information (enrolled population).

	Patients (*n* = 255)
Concomitant procedures, *n* (%)	
Annular debridement	76 (29.8)
Ascending aorta replacement	72 (28.2)
Coronary artery bypass grafting	36 (14.1)
Aortic aneurysm/dissection repair	35 (13.7)
Root sinus enlargement/aortic annular enlargement	29 (11.4)
Atrial septal defect repair	7 (2.7)
Unplanned procedures	6 (2.4)
Atrial ablation	5 (2.0)
Automated implanted cardioverter implant	1 (0.4)
Intra‐aortic balloon pump use	1 (0.4)
Other procedures^a^	56 (22.0)
Surgical approach, *n* (%)	
Full sternotomy	178 (69.8)
Mini upper sternotomy	22 (8.6)
Right thoracotomy	3 (1.2)
Other^b^	52 (20.4)
Valve size (mm), *n* (%)	
19	6 (2.4)
21	20 (8.0)
23	71 (28.4)
25	127 (50.8)
27	26 (10.4)
29	0
Aortic cross‐clamp time (min), mean ± SD	84.8 ± 30.40
Extracorporeal circulation duration (min), mean ± SD	118.0 ± 42.14
Procedural duration (min), mean ± SD	245.7 ± 73.35

^a^ Other procedures include ligation of patent ductus arteriosus, suture and ligation of the left atrial appendage, left atrial appendage resection, partial aortic arch artificial vessel replacement, aortic arch replacement, ascending aortoplasty, left ventricular outflow duct dredging, dissection of cervical and inguinal lymph nodes, tricuspid valvuloplasty (Kay's), mitral valve repair, septal repair, and cautery division of pleural adhesions.

^b^ Other surgical approaches include minimally invasive thoracoscopic aortic valve replacement and right parasternal intercostal incision.

### 3.3 Clinical Outcomes and Complications

**Figure 2:**
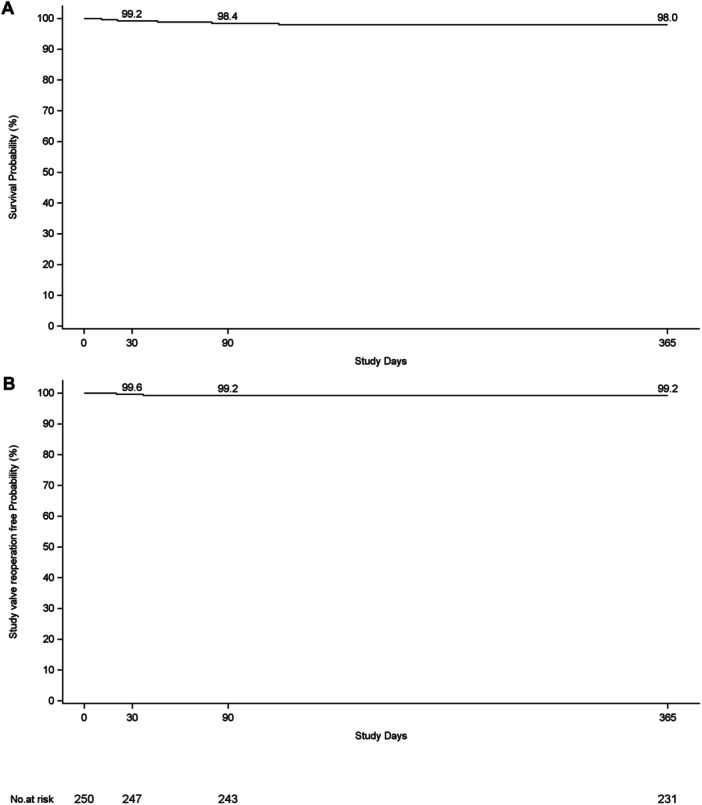
Kaplan−Meier curve of 1‐year freedom from all‐cause death (A) and reoperation on the study valve (B) in patients undergoing aortic valve replacement with the study valve.

Of the 250 patients, the freedom from all‐cause death rate was 99.2% at 30 days and 98.0% at 1 year. The 1‐year freedom from cardiovascular death was 98.8% (3 deaths: 1 from cardiac arrest, 1 from heart failure, and 1 from acute ischemic stroke). Two additional deaths resulted from septic shock (Figure 2). Detailed descriptions of death cases are summarized in Supporting Information S1: Table 1. All deaths were assessed by investigators as unrelated to the device.

The 1‐year freedom from reoperation on the study valve was 99.2% (2 reoperations observed). The first case required explantation of the study valve on postoperative Day 19 due to infective endocarditis. In the second case, the study valve was re‐sutured successfully on postoperative Day 37 due to PVL. The event was assessed by the investigators as unrelated to the study valve and attributed to the patient's underlying autoimmune disease (Behçet's disease).

Thromboembolic events occurred in 2.4% of patients (5 strokes and 1 transient ischemic attack). Major bleeding was observed in 11 patients (4.4%). Eight cases occurred within 30 days postoperatively, originating from the pericardium (6 cases) and mediastinum (2 cases). Six bleeding events (4 pericardial, 2 mediastinal) required re‐thoracotomy for exploration, while 2 patients with pericardial hemorrhage were treated with pericardial puncture. Seven of these events were assessed as related to the surgical procedure, and the other one was considered related to warfarin intolerance by the investigator. The remaining 3 cases occurred beyond 30 days postoperatively and consisted of gastrointestinal tract bleeding (2 cases) and intracerebral bleeding (1 case). Hemostasis was achieved in all major bleeding cases. None of the major bleeding events were related to the study valve itself. Of all major bleeding and thromboembolic events, 5 major bleeding events (including an intracerebral hemorrhage, which was also classified as a thromboembolic event) were deemed attributable to antithrombotic therapy; none of these patients had other underlying conditions requiring antithrombotic therapy (Supporting Information S1: Table 2).

One patient (0.4%) received a permanent pacemaker. One patient (0.4%) developed infective endocarditis 11 days postoperatively, which resolved completely after antibiotic treatment and valve explantation (Table 3). No valve‐related death, SVD, non‐cerebral thromboembolism, or valve thrombosis events were reported (Table 3).

**Table 3:** Safety outcomes (valve‐implanted population).

	30 days		3 months		1 year		1‐year freedom from event (%) (95% CI)
	Patients, *n* (%)	Patients at risk, *n*	Patients, *n* (%)	Patients at risk, *n*	Patients, *n* (%)	Patients at risk, *n*	
All‐cause death	2 (0.8)	248	4 (1.6)	243	5 (2.0)	231	98.0 (95.2, 99.2)
Cardiovascular death	1 (0.4)	248	2 (0.8)	243	3 (1.2)	231	98.8 (96.3, 99.6)
Valve‐related death	0	248	0	243	0	231	100 (100, 100)
Structural valve deterioration	0	248	0	243	0	231	100 (100, 100)
Reoperation on the study valve	1 (0.4)	247	2 (0.8)	243	2 (0.8)	231	99.2 (96.8, 99.8)
Valve explant	1 (0.4)	247	1 (0.4)	243	1 (0.4)	231	99.6 (97.2, 99.9)
Major PVL^a^	1 (0.4)	247	1 (0.4)	243	1 (0.4)	231	99.6 (97.2, 99.9)
Nonstructural valve dysfunction (other than PVL)	0	248	0	243	0	231	100 (100, 100)
Major bleeding	8 (3.2)	240	8 (3.2)	237	11 (4.4)	222	95.6 (92.1, 97.5)
Thromboembolic event	5 (2.0)	243	5 (2.0)	238	6 (2.4)	227	97.6 (94.7, 98.9)
Stroke	4 (1.6)	244	4 (1.6)	239	5 (2.0)	228	98.0 (95.2, 99.2)
Transient ischemic attack.	1 (0.4)	247	1 (0.4)	242	1 (0.4)	230	99.6 (97.2, 99.9)
Non‐cerebral thromboembolism	0	248	0	243	0	231	100 (100, 100)
Valve thrombosis	0	248	0	243	0	231	100 (100, 100)
Permanent pacemaker implant	1 (0.4)	247	1 (0.4)	242	1 (0.4)	230	99.6 (97.2, 99.9)
Endocarditis	1 (0.4)	247	1 (0.4)	243	1 (0.4)	231	99.6 (97.2, 99.9)

Abbreviation: PVL, paravalvular leak.

^a^ Major PVL is PVL of any grade requiring surgical intervention or considered a serious adverse event.

**Figure 3:**
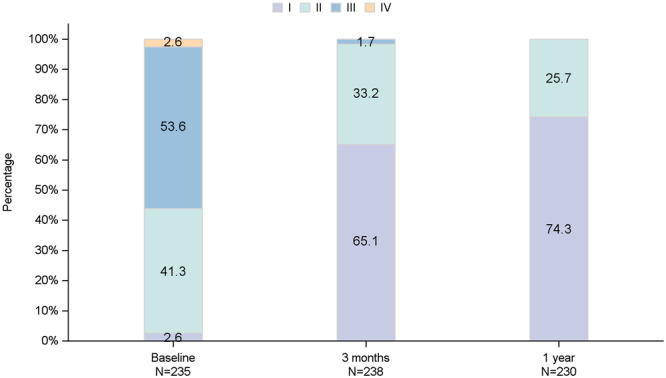
New York Heart Association heart failure classification during follow‐up (valve‐implanted population).

Patients demonstrated functional improvements as measured by NYHA Class, with 74.3% of 230 patients assessed as Class I and 25.7% as Class II at 1 year (Figure 3).

**Figure 4:**
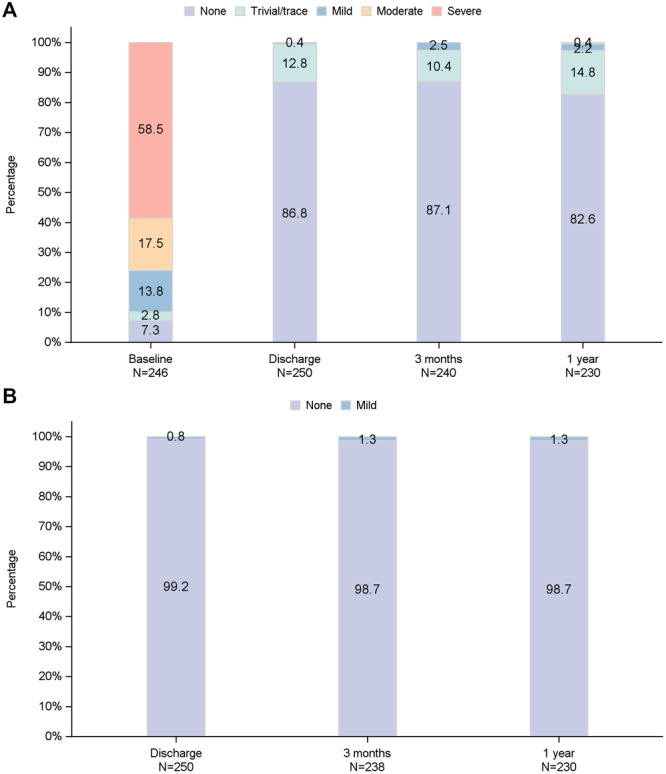
Aortic regurgitation and valvular regurgitation during follow‐up (valve‐implanted population). (A) Aortic regurgitation, (B) Paravalvular Leak.

At 1‐year post‐procedure, no patients had severe aortic regurgitation (AR); 1 patient (0.4%) developed asymptomatic moderate AR. Three cases (1.3%) of PVL were observed, all mild in severity (Figure 4). Hemodynamic assessments are shown in Supporting Information S1: Figure 2 (stratified by visit) and Supporting Information S1: Figures 3 and 4 (stratified by visit and valve size). The mean EOA and the EOA index at the 1‐year follow‐up were 1.7 ± 0.59 cm^2^ and 0.9 ± 0.31 cm^2^/m^2^, respectively, and the mean transvalvular pressure gradient was 11.7 ± 5.21 mmHg, which remained stable within 1 year after AVR.

## 4 Discussion

This post‐market, real‐world clinical study evaluated the safety and effectiveness of the INSPIRIS RESILIA aortic valve in the Chinese population undergoing elective AVR. As the first post‐market investigation of this device in a real‐world Chinese cohort, this study addresses a critical evidence gap by providing insights into routine practice. The results demonstrated the 1‐year freedom from all‐cause death of 98.0%, with all deaths assessed as unrelated to the study device. Importantly, no SVD occurred, and hemodynamics remained stable, aligning with early outcomes of the COMMENCE trial [20].

This real‐world study of Chinese AVR patients revealed a younger cohort (58.5 ± 9.17 years) compared to the COMMENCE trial (67.0 ± 11.6 years) [20]. Additionally, the baseline disease severity was greater in the current cohort (49.8% NYHA Class III, 2.4% Class IV). These proportions exceed those reported in the COMMENCE trial (24.4% Class III, 1.9% Class IV) and the INDURE registry (patients < 60 years old; 27.2% Class III/IV) [20, 21]. Concomitant cardiac procedures, particularly ascending aortic replacement (28.9%) and root sinus/aortic annular enlargement (11.4%), were more common than in the COMMENCE trial (ascending aorta replacement 1.2%, root sinus enlargement 2.2%, annular enlargement 1.3%) or the INDURE registry (root replacement 1.4%), reflecting prevailing clinical practices that incorporate multiple interventions in a single operative session.

Despite baseline differences, the study valve demonstrated a favorable safety profile. Freedom from all‐cause mortality was 99.2% at 30 days and 98.0% at 1 year, with no valve‐related deaths. Comparable early (≤ 30 days) outcomes were demonstrated in the COMMENCE trial (98.8% freedom from all‐cause death; 99.6% freedom from valve‐related death), with corresponding 1‐year rates of 97.6% and 98.8%, respectively [20]. Similarly, the INDURE registry showed a 99.3% and 98.3% freedom from all‐cause death at 30 days and 1 year, respectively, with no study valve‐related deaths [21]. In contrast, the 1‐year all‐cause death rate for other conventional bioprosthetic valves ranges from 7.6% to 13.1% [22, 23, 24]. No patients remained in NYHA Classes III or IV at 1 year in the current study. This functional improvement was numerically better compared with the COMMENCE trial (1.6% Class III, 0.4% Class IV at 1 year) and the INDURE registry (3.3% Class III, 0.3% Class IV at 1 year) [20, 21]. These disparities may potentially be attributed to the higher proportion of patients receiving root sinus enlargement, as larger valve sizes may enhance exercise tolerance following AVR [25, 26, 27]. Additionally, the current study demonstrated a 1‐year freedom from permanent pacemaker implantation rate of 99.6%, exceeding the corresponding rates of 94.5% in the COMMENCE trial and 95.4% in the INDURE registry [20, 21]. However, the association between concomitant root enlargement and the incidence of permanent pacemaker implantation remains controversial [25, 28]. Previous research suggests that concomitant root enlargement with AVR is not an independent risk factor of pacemaker implantation compared to isolated AVR [25]. Collectively, these findings support the utility of the study valve in improving AVR outcomes and its applicability to broader patient populations.

Anatomical differences in the aortic root, particularly smaller annulus areas in Asian compared with Caucasian patients, should be considered when interpreting the results [29]. Prior studies indicate racial variations in the distribution of implanted valve sizes. The Trifecta valve study in North America reported size distributions [30] contrasting with a trend towards smaller sizes in Japan [31]. However, the difference in valve size distributions was not as pronounced in Chinese patients in the current study compared with the COMMENCE trial results from North America and Europe [20] (Supporting Information S1: Table 3). This may be attributed to a rate of concomitant root sinus enlargement or aortic annular enlargement procedures (11.4%) in the current study compared to the COMMENCE trial (2.2% and 1.3%, respectively), despite a lower prevalence of obesity (5.5% with BMI ≥ 30 kg/m² vs. 43.7% in COMMENCE) [20]. Notably, among patients undergoing root sinus enlargement, the majority received larger valves (25 or 27 mm), with only 1 patient receiving a 21‑mm valve and no 19‑mm valves being used. The observed trend toward implanting larger valve prostheses in AVR may enhance post‐procedural hemodynamic performance following AVR [26], potentially optimizing valve lifetime management strategies to maximize valve durability and mitigate risks of valve failure or reoperation on the study valve.

The INSPIRIS RESILIA aortic valve technology is distinguished by its advanced anti‐calcification properties. Clinically, these anti‐calcification properties may enhance valve durability, particularly among younger patients. The present study reported no SVD events, consistent with early results from the COMMENCE trial [20]. However, this study has several limitations. The 1‐year follow‐up is insufficient to evaluate long‐term durability. While the present study provides only early (1‐year) safety and hemodynamic data, previous clinical studies of the study valve, including the COMMENCE trial, have demonstrated excellent durability with 99.3% freedom from SVD and stable valve performance at 8 years of follow‐up [13]. Durability of the INSPIRIS valve in the Chinese population still requires long‐term follow‐up. In addition, the outcome definitions in this study were based on earlier consensus criteria [18, 19] rather than the more recent Valve Academic Research Consortium 3 (VARC‐3) framework [32], consistent with the prospective design aligned with the multinational COMMENCE trial. This methodology applied the surgical valve replacement acceptance criteria (objective performance criteria), which were developed based on the Akins definitions [19] and have been referenced in the International Organization for Standardization (ISO) standards [33]. While this alignment facilitates comparison with established data from the COMMENCE trial, it may limit direct comparability with contemporary studies using VARC‐3 definitions. Nevertheless, by incorporating comprehensive imaging and hemodynamic assessments, the current study ensured the capture of key valve‐related events. Long‐term follow‐up of this cohort is ongoing, and future reports will provide extended data on durability, SVD, and clinical outcomes.

## 5 Conclusion

This prospective multicenter study provides early evidence supporting the favorable 1‐year safety and effectiveness profile of the INSPIRIS RESILIA aortic valve in a Chinese population. The observed outcomes included low death rates, sustained hemodynamic stability, and absence of SVD events at 1 year. Notably, a trend emerged towards implanting larger valves with concomitant aortic root enlargement, which could improve long‐term outcomes and support comprehensive lifelong management. Collectively, these findings position this bioprosthesis as a promising therapeutic option.

## Author Contributions

**Haitao Xu:** conceptualization, methodology, visualization, writing – original draft. **Yang Yan:** data curation, investigation, supervision, writing – review and editing. **Xin Chen:** data curation, investigation, validation, writing – review and editing. **Jiangang Wang:** data curation, investigation, writing – review and editing. **Hansong Sun:** conceptualization, data curation, investigation, methodology, project administration, visualization, writing – review and editing.

## Ethics Statement

The study adhered to the Declaration of Helsinki and local regulations. Ethical approval was obtained from the institutional review board at each participating center (Supporting Information Materials). Written informed consent was obtained from all patients.

## Conflicts of Interest

Authorship and manuscript composition reflect cooperation among multiple investigators, sites, and Edwards Lifesciences. All authors have nothing to disclose with regard to commercial support.

## Supporting information

Supporting File

## Data Availability

The data underlying this article are owned by Edwards Lifesciences LLC and Edwards (Shanghai) Medical Product Co. Ltd. Data will be shared on reasonable request to the corresponding author with permission of Edwards Lifesciences LLC and Edwards (Shanghai) Medical Product Co. Ltd. The data that support the findings of this study are available on request from the corresponding author. The data are not publicly available due to privacy or ethical restrictions.
