# Supplementary material for: Early Outcomes of Real‐World Aortic Valve Replacement With RESILIA Tissue in the Chinese Population
Source: Clin Cardiol. 2026 Jun 15;49(6):e70347. doi: 10.1002/clc.70347 (PMC13267994; doi:10.1002/clc.70347)
Supplement: Supplementary file 1 — Supporting File [file CLC-49-e70347-s001.docx]

**Supplementary materials**

**Ethics approval center and approval number**

| **Ethics Approval unit** | **Ethical approval No.** |
| --- | --- |
| Fuwai Hospital, Chinese Academy of Medical Sciences and Peking Union Medical College | 2022-1840 |
| Fuwai Yunnan Cardiovascular Hospital | 2021-073-01 |
| Xiamen Cardiovascular Hospital Xiamen University | (2022)医伦第(8)号 |
| Nanjing First Hospital | KY20220321-01-KS-01 |
| The First Affiliated Hospital of Zhengzhou University | L2022-Q049-003 |
| Fuwai Hospital Chinese Academy of Medical Sciences, Shenzhen | SP2022070(01) |
| The Frist Affiliated Hospital of XI'an Jiaotong University | X-XJTU1AF2022LSY-203 |
| Fuwai Central China Cardiovascular Hospital | 2022-Q004-02 |
| Beijing Anzhen Hospital, Capital Medical University | (2022)器伦审第(44)号 |
| The First Affiliated Hospital of Sun Yat-sen University | 伦审临[2022]076号 |
| Sichuan Provincial People's Hospital | 伦审(械) 2022年第16-1号 |
| Henan Provincial Chest Hospital | (2023)科伦审第(04-03)号 |

**Supplementary Figure S1.** Flowchart by visit

**
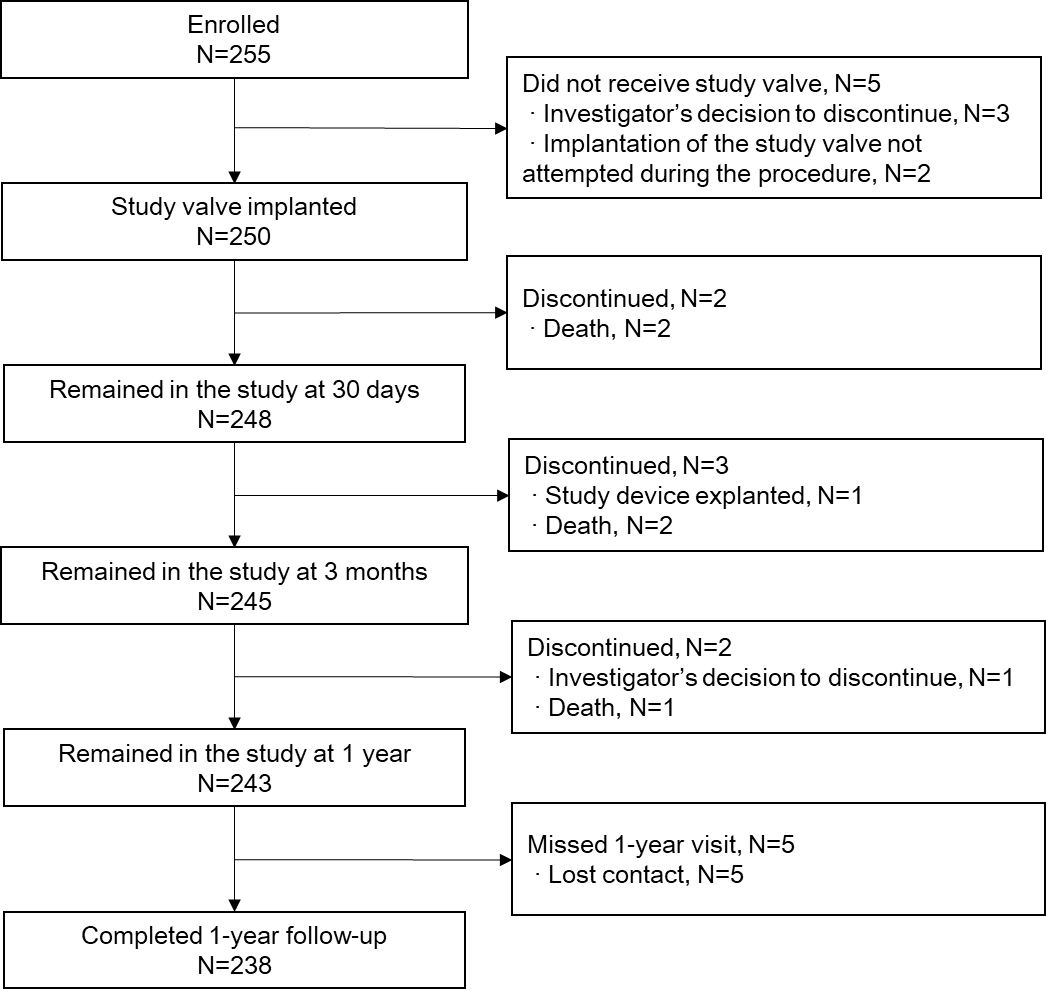
**

**Supplementary Figure S2****.** Effective Orifice Areas and Mean Pressure Gradients by Visit (valve-implanted population).


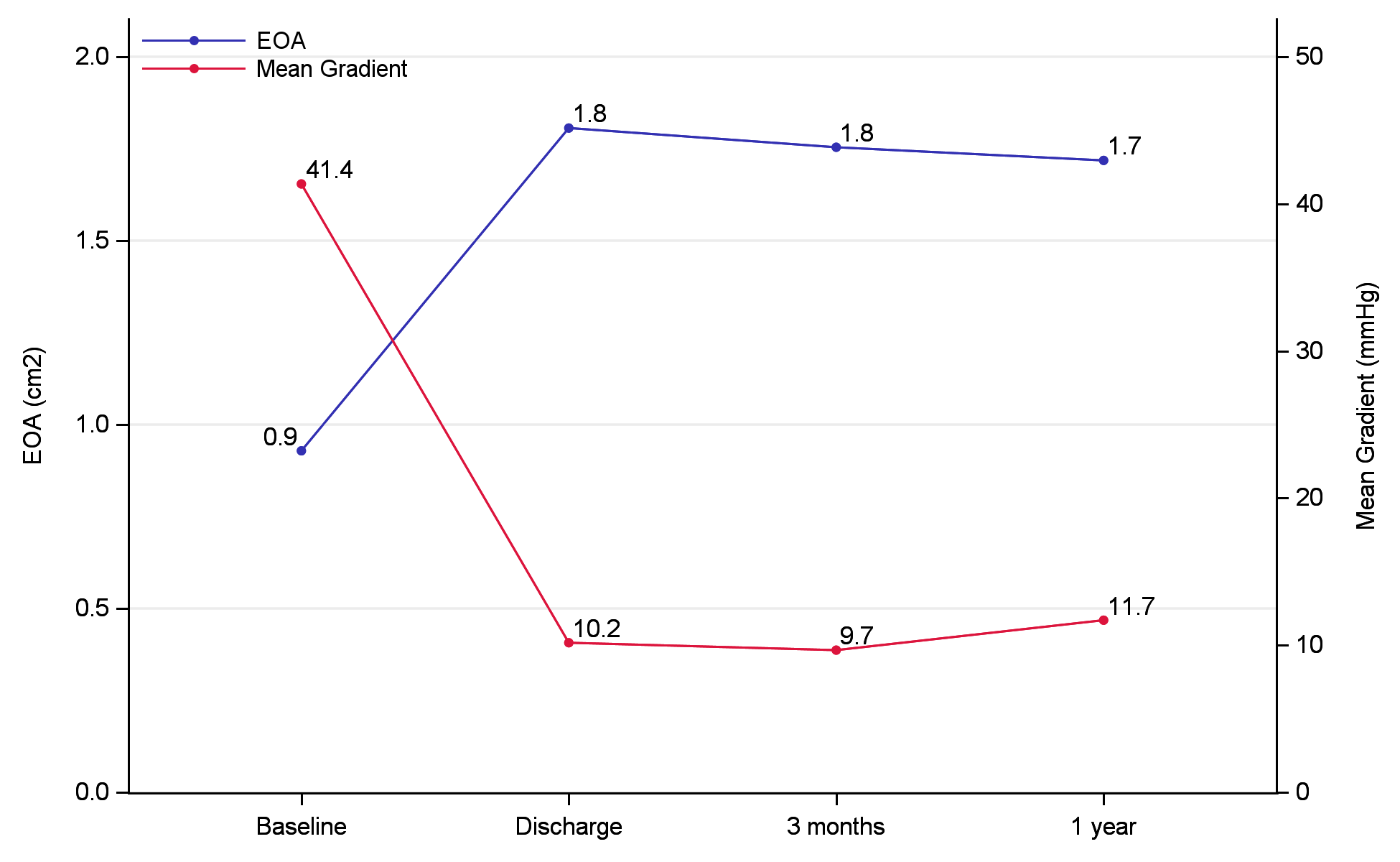


EOA: effective orifice area.

**Supplementary Figure S3.** Effective Orifice Areas by Visit and Valve Size (valve-implanted population)


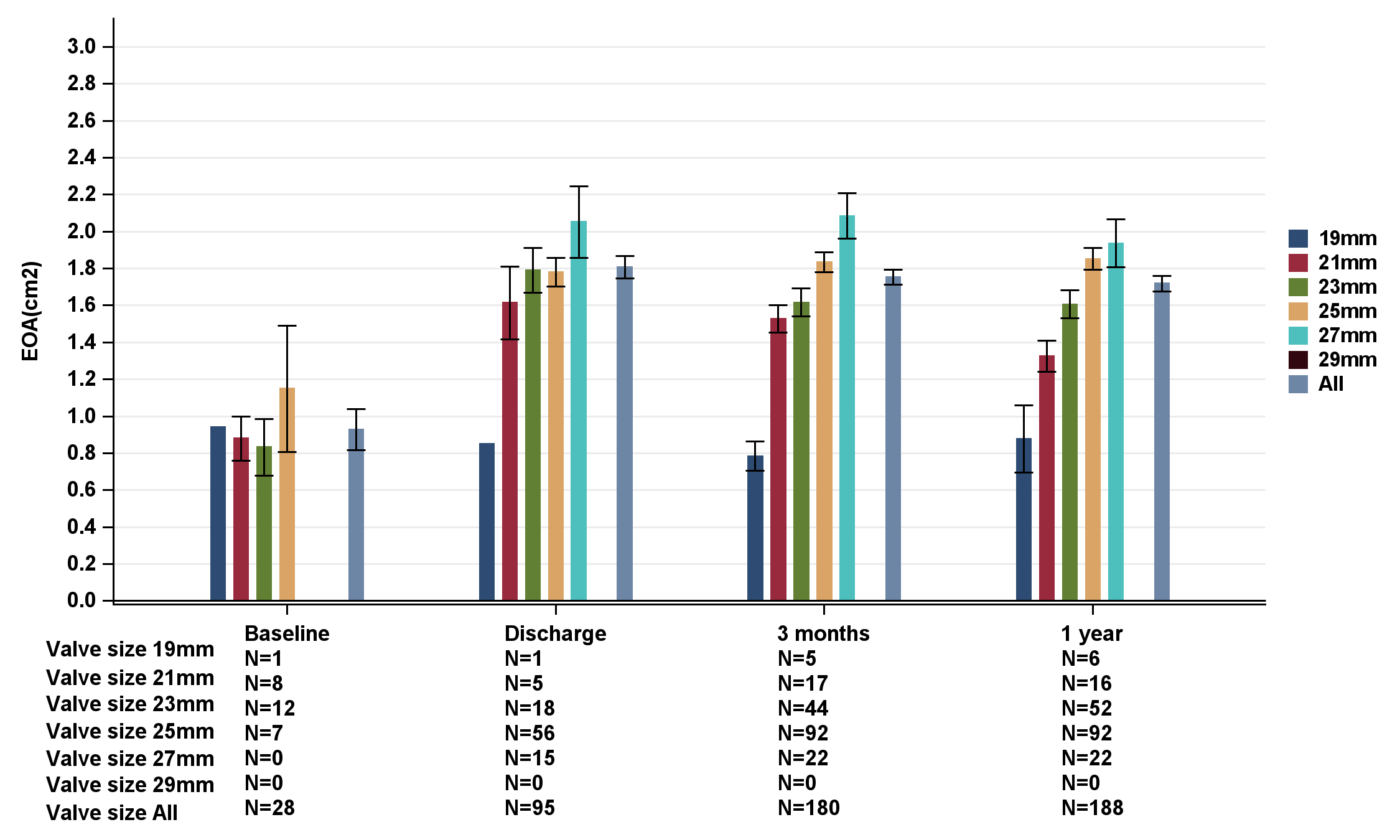


EOA: effective orifice area.

**Supplementary Figure S4.** Mean Gradient by Visit and Valve Size (valve-implanted population)


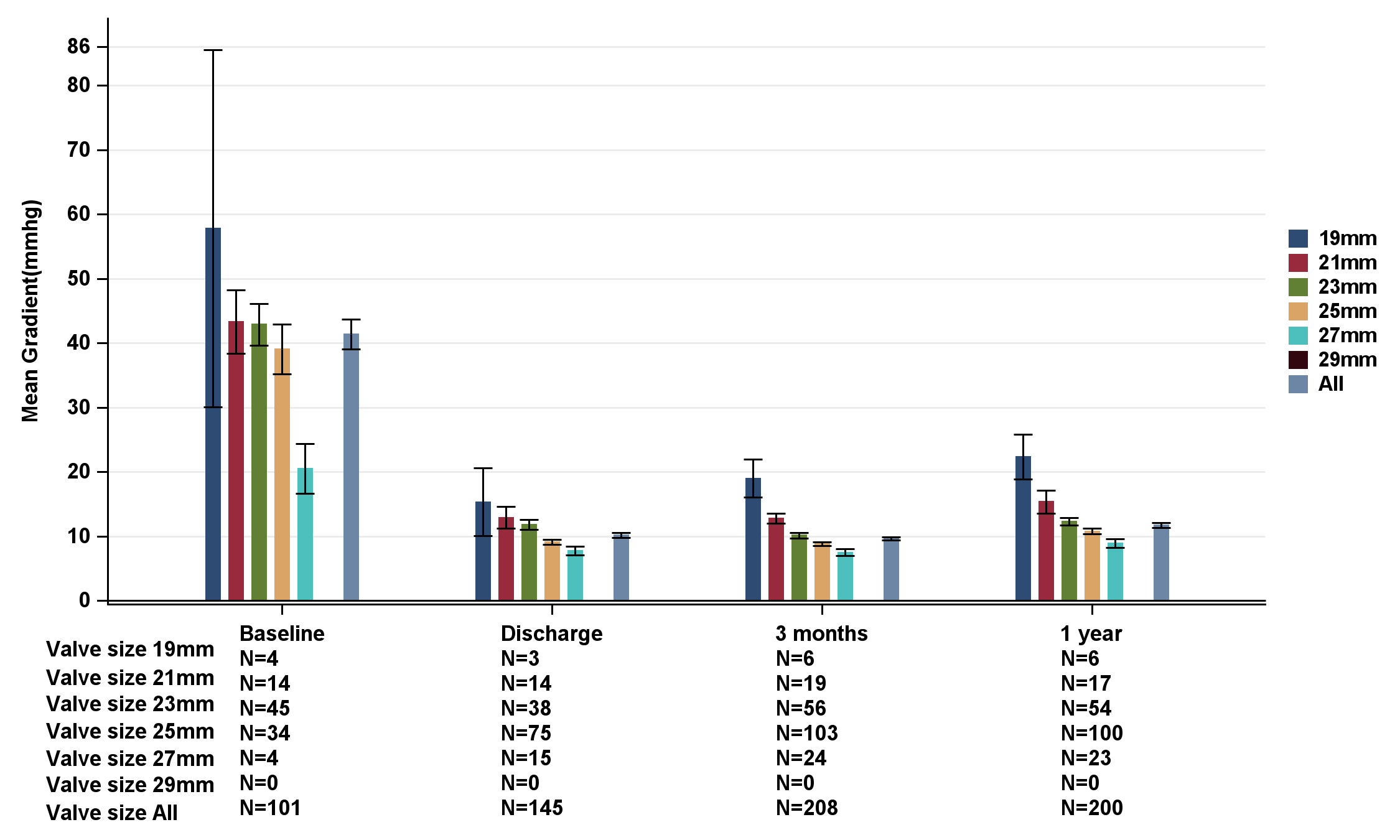


**Supplementary Table S1.** Description of death cases

| **Case** | **Valve size** | **Death date** | **Study day of Death** | **Categorical cause of Death** | **Primary reason for death** | **Study valve related death** | **Adverse events term that resulted in death** |
| --- | --- | --- | --- | --- | --- | --- | --- |
| 1 | 23mm | 2024-01-10 | 22 | Non-cardiovascular |  | No | Infection/Inflammation - Other |
| 2 | 25mm | 2023-05-05 | 81 | Cardiovascular | Heart failure | No | NSD - Paravalvular leak +4 Major |
| 3 | 25mm | 2023-08-04 | 123 | Cardiovascular | Stroke | No | Thromboembolic event – Stroke |
| 4 | 23mm | 2023-01-06 | 47 | Non-cardiovascular |  | No | Sternal wound/Thoracic infection |
| 5 | 25mm | 2023-09-24 | 12 | Cardiovascular | Cardiovascular Hemorrhage | No | Cardiac arrest |

**Supplementary Table S2.** Adverse events considered related to antithrombotic therapy

| **Case** | **Adverse events** | **Post-operation day of the event** | **Medical history requiring antithrombotic therapy** | **Antihrombotic therapy at the time of AE** |
| --- | --- | --- | --- | --- |
| 1 | Pericardial hemorrhage | 20 | No | Warfarin |
| 2 | Pericardial hemorrhage | 21 | No | Warfarin |
| 3 | Intracerebral hemorrhage | 158 | No | Warfarin |
| 4 | Acute duodenal bulb ulcer with bleeding | 150 | No | Warfarin |
| 5 | Duodenal ulcer with bleeding | 167 | No | Clopidogrel |

**Supplementary Table S3.** Distribution of the implanted size valves across different populations

| **Study** | **Goldman et al., 2017 ^1^** | **Tadokoro et al., 2018 ^2^** | | **COMMENCE trial, Puskas et al., 2017 ^3^** | **The present study** |
| --- | --- | --- | --- | --- | --- |
| Population | North America | Japan | | North America and Europe | China |
| Study valve | Trifecta | Trifecta | Magna | RESILIA | RESILIA |
| Sizes (mm), % |  |  |  |  |  |
| 19 | 11.1 | 35.0 | 25.8 | 3.2 | 2.4 |
| 21 | 27.6 | 23.3 | 31.5 | 19.0 | 8.0 |
| 23 | 33.1 | 34.0 | 25.8 | 31.1 | 28.4 |
| 25 | 21.4 | 7.8 | 11.0 | 29.3 | 50.8 |
| 27 | 5.4 | 0 | 5.9 | 14.5 | 10.4 |
| 29 | 1.4 | 0 | 0 | 2.9 | 0 |

**References**

1. Goldman S, Cheung A, Bavaria JE, Petracek MR, Groh MA, Schaff HV: Midterm, multicenter clinical and hemodynamic results for the Trifecta aortic pericardial valve. *J Thorac Cardiovasc Surg*. 2017;153:561-569.e562.

2. Tadokoro N, Fukushima S, Shimahara Y, et al.: Trifecta vs. Magna for Aortic Valve Replacement- Differences in Clinical Outcome and Valve Hemodynamics. *Circ J*. 2018;82:2767-2775.

3. Puskas JD, Bavaria JE, Svensson LG, et al.: The COMMENCE trial: 2-year outcomes with an aortic bioprosthesis with RESILIA tissue†. *Eur J Cardio-Thorac Surg*. 2017;52:432-439.
